# Proximal tibiofibular dislocation: a case report of this often overlooked injury

**DOI:** 10.1259/bjrcr.20150372

**Published:** 2016-07-28

**Authors:** Benjamin Martin, James Corbett, Alastair Littlewood, Rupert Clifton

**Affiliations:** ^1^Core Surgical Trainee, East of England Deanery, UK; ^2^Specialist Registrar in Trauma and Orthopaedics, East of England Deanery, UK; ^3^Department of Radiology, Peterborough City Hospital, Peterborough, UK; ^4^Department of Trauma and Orthopaedics, Peterborough City Hospital, Peterborough, UK

## Abstract

Tibiofibular dislocation is a condition that is a recognized cause of lateral knee pain in trauma patients and can occur in isolation or as a part of multiple injuries. There is usually prominence of the fibular head on clinical examination, with tenderness to palpation. Radiological investigation can confirm the diagnosis, and in the case or our patient, both plain radiographs and MRI were performed. MRI permitted pre-reduction assessment of the intrinsic knee ligaments, as well as the common peroneal nerve. The dislocated fibular head was successfully relocated under general anaesthesia as a closed reduction.

## Clinical presentation

A 29-year-old male, who was normally fit and well, presented to the emergency department following a fall playing football. The patient had stood on the ball, with the ball rolling under his weight and twisting his knee.

On examination, there was a prominence over the lateral aspect of the proximal left leg. He was exquisitely tender over the fibular head, with no obvious knee effusion. He was unable to weight bear but was neurovascularly intact, with no features of common peroneal nerve injury. His ankle joint was stable and non-tender, with no suggestion of disruption of the distal tibiofibular joint.

**Figure 1.**
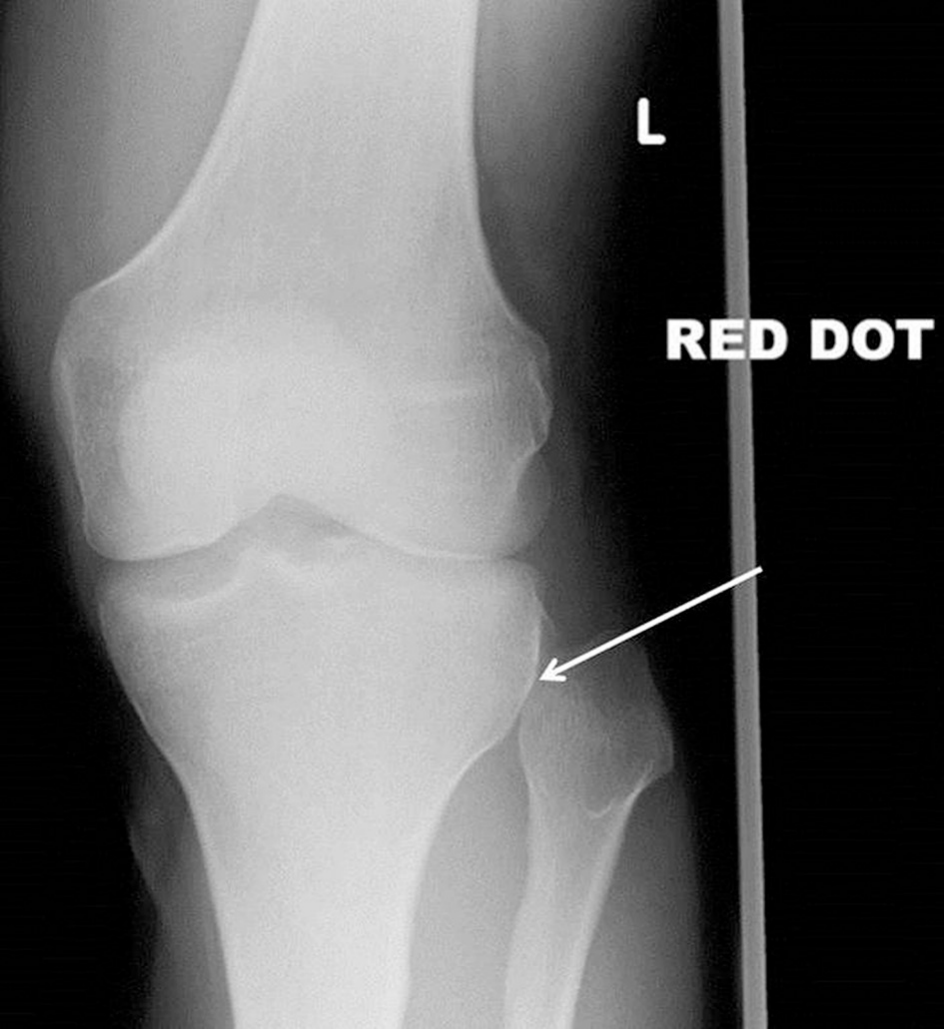
Anteroposterior radiograph of the patient’s left knee showing lateral tibiofibular dislocation. The arrow highlights the lack of tibiofibular overlap. “Red dot” indicates an abnormality identified by the radiographer

**Figure 2.**
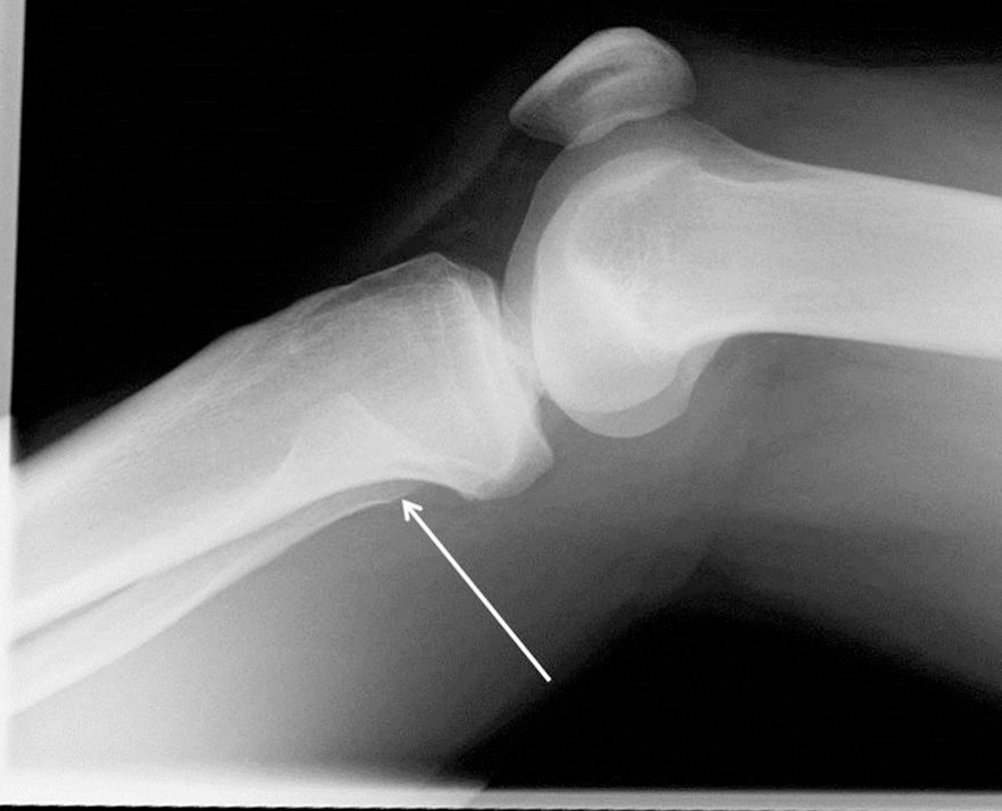
Lateral radiograph of the patient’s left knee showing anterior tibiofibular dislocation. The arrow indicates the direction of dislocation in this view.

Plain radiographs of the knee were suggestive of proximal tibiofibular dislocation, with no overlap of the fibula and tibia on the anteroposterior (AP) film (Figure 1), and anterior displacement of the fibular head on the lateral film (Figure 2). Radiographs of the ankle confirmed no distal tibiofibular injury.

**Figure 3.**
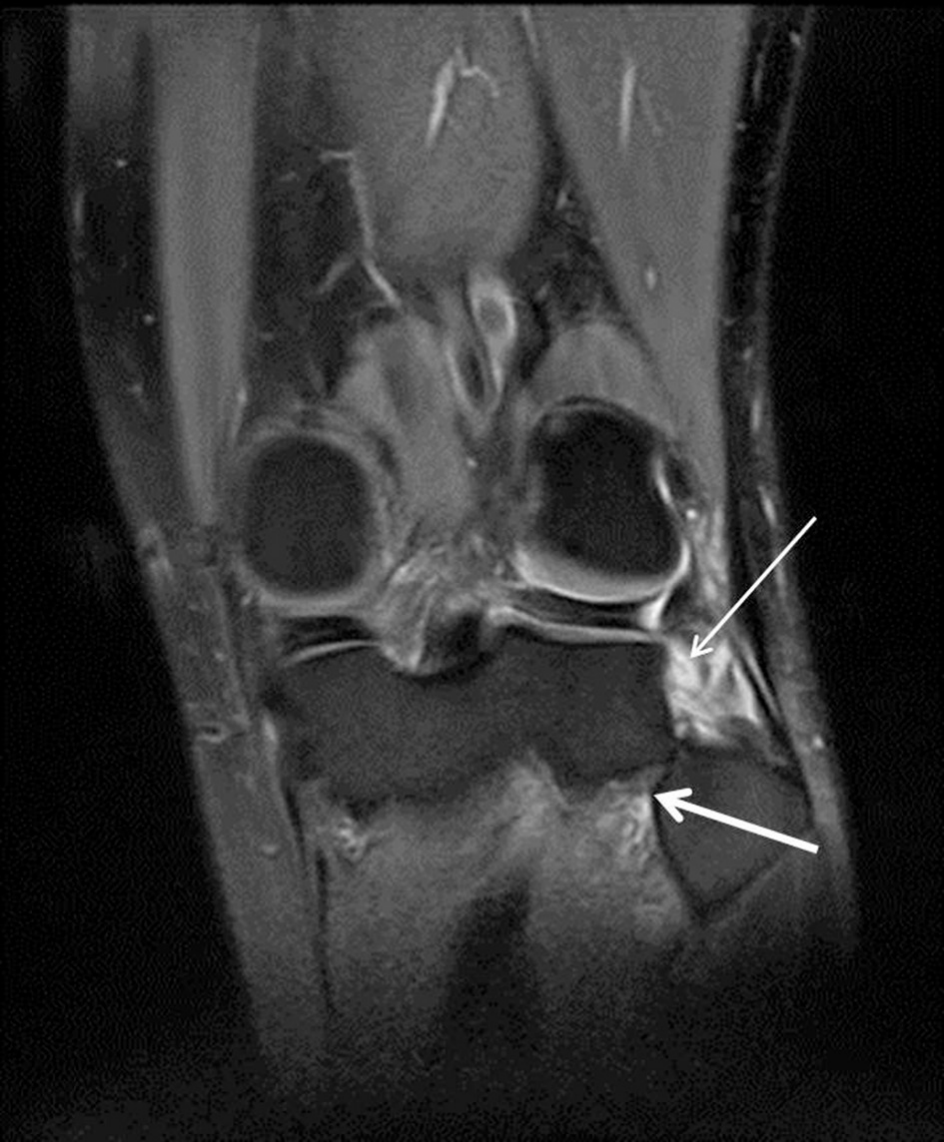
Coronal proton density fat saturation MRI [repetition time (TR) = 4500/echo time (TE) = 32.18] of the left knee showing high signal around the tibiofibular ligament complex (thin arrow) and lateral translation of the fibular head (thick arrow).

**Figure 4.**
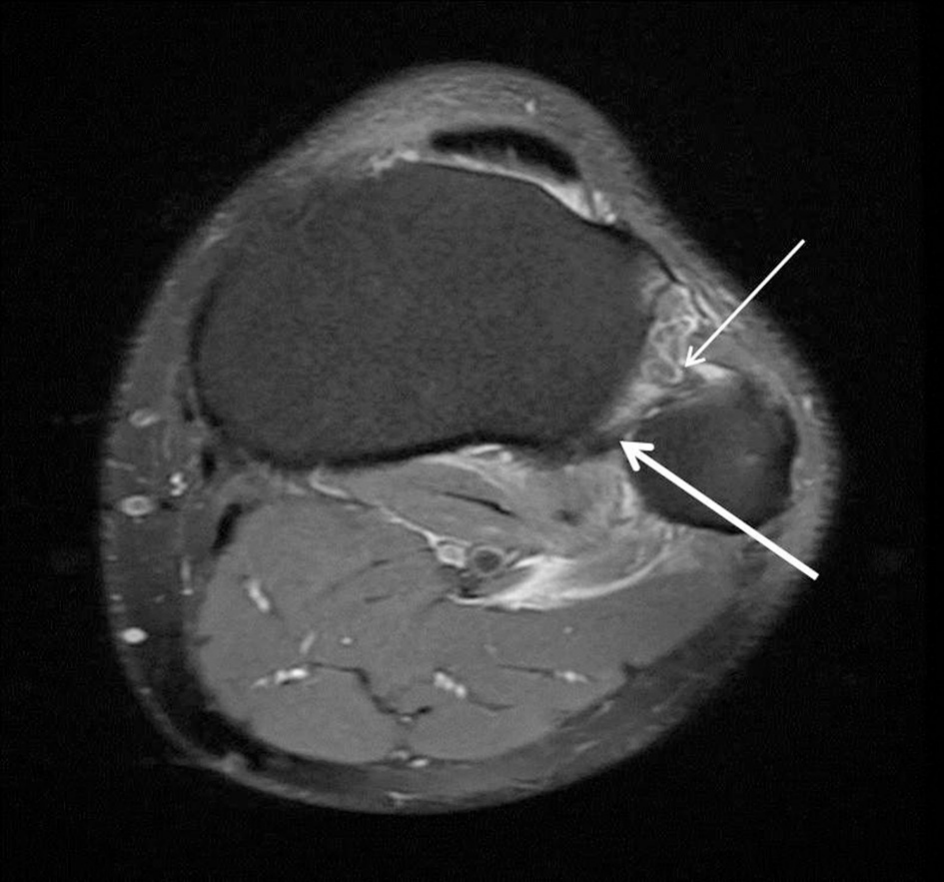
Axial proton density fat saturation MRI [repetition time(TR) = 3480/echo time (TE) = 31.87] of the left knee showing high signal around the tibiofibular ligament complex (thin arrow) and anterolateral translation of the fibular head (thick arrow).

MRI of the knee was undertaken to confirm the diagnosis and exclude ligamentous injury; this confirmed anterolateral tibiofibular dislocation (Figures 3 and 4).

It also permitted us to further assess the common peroneal nerve, which can be injured in posterior fibular dislocation. The report notes:

The proximal fibula is dislocated anterolaterally from its usual position, with extensive high signal around the tibiofibular ligament.
There is diffuse high signal around the lateral collateral ligament with no significant disruption of the ligament itself.
Popliteus is intact. No injury to the common peroneal nerve is seen.
No fracture or tendon injury is demonstrated, and the remainder of the internal structures of the knee appear normal."

**Figure 5.**
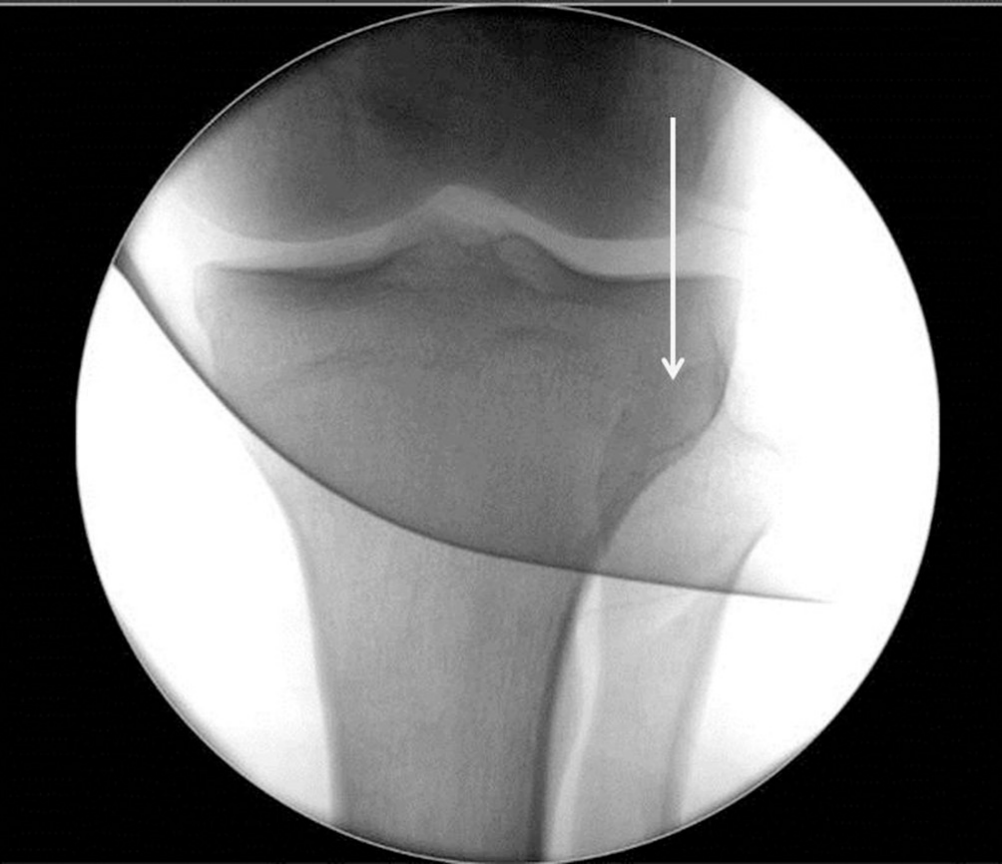
Anteroposterior post-reduction image intensifier image of the left knee showing normal tibiofibular overlap. Arrow indicates the area of tibio-fibular overlap.

The patient was taken to the operation theatre for closed reduction under general anaesthesia, with the reduction visualized under the image intensifier (Figure 5), confirming a satisfactory outcome. The patient was discharged in an above-knee backslab for 2 weeks, non-weight bearing. Weight-bearing ability would be expected to improve significantly after this point.

## Discussion

The proximal tibiofibular joint is a synovial joint that consists of the lateral tibial condyle and the fibular head. They articulate within a fibrous capsule and are supported by anterosuperior and posterosuperior tibiofibular ligaments.^1^

Proximal tibiofibular dislocation (PTFD) is a condition first recognized and reported by Nelation^2^ in 1874 and has continued to be an uncommon condition for which the clinician should have a high index of suspicion. It can happen in isolation or in the context of a patient with multiple injuries.

A recent review^3^ notes that PTFD occurs in 1–2% of tibial shaft/plateau fractures, and is a marker of severe lower limb trauma. 31 cases of isolated PTFD have been published since 1974,^4,5^ which have occurred following a wide range of mechanisms,^6,7^ ranging from football^8^ to long jump,^9^ falling from a height^10^ to snowboarding.^11^ The injury occurs in flexion when anatomically the lateral collateral ligamentous support is removed. *Rockwood and Green’s fractures in adults*^12^ notes that isolated injuries, as in the case of our patient, usually arise from sporting injuries caused by exaggerated twisting.

**Figure 6.**
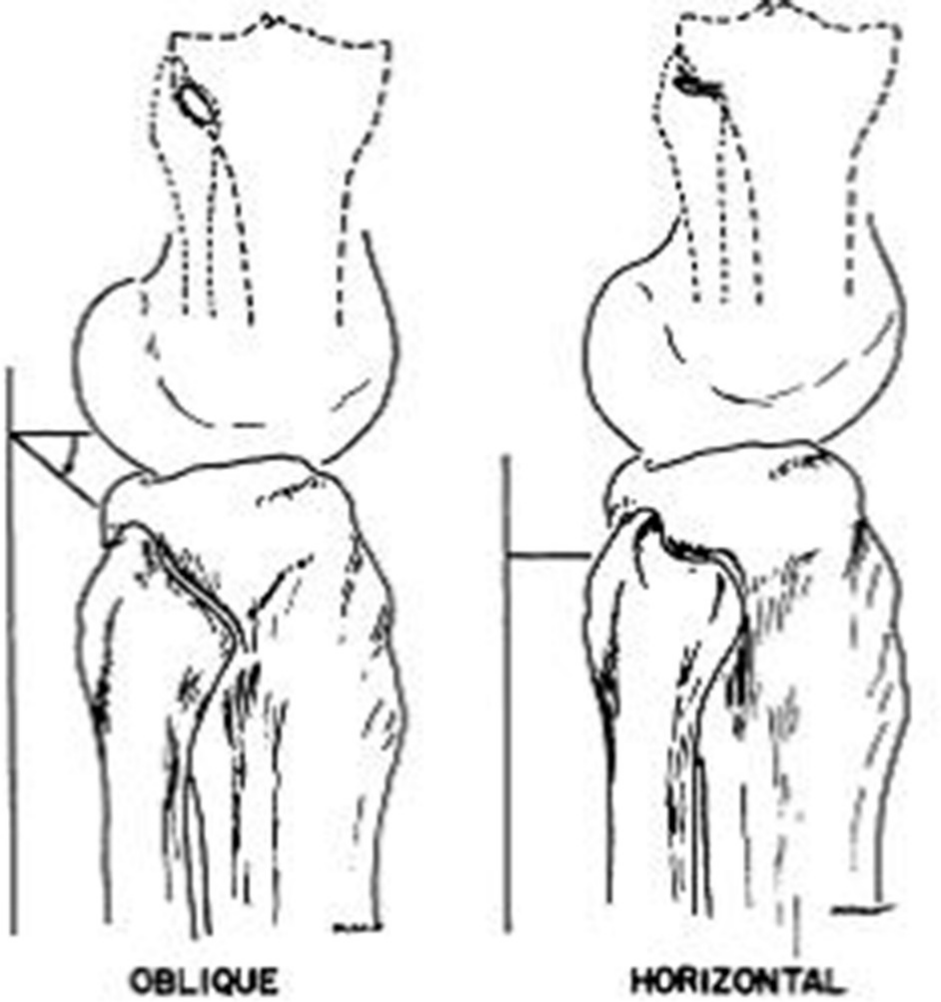
The two different tibiofibular joint structures. Reproduced from Ogden^13^ 1974 with permission from Rockwater Inc.

Four recognized patterns of injury to this joint have been noted.^13,14^ The least severe is subluxation, and there are three directions of dislocation (anterolateral, superior and posteromedial). Ogden^13^ also noted two different anatomical joint structures (oblique or horizontal, Figure 6), with the oblique variant having less articular surface area.

Imaging is usually diagnostic on plain radiographs, and this can be made easier by appropriate patient positioning and imaging both knees to facilitate comparison. Diagnostic yield is 72% on plain radiographs and improves to 82% with control images to compare with.^15^

**Figure 7.**
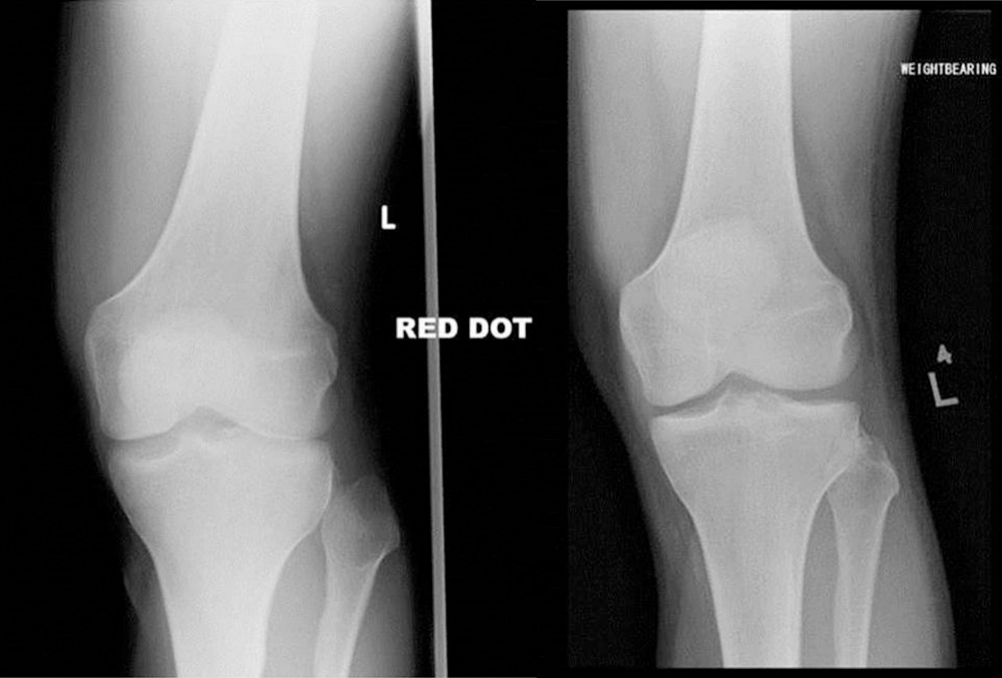
Anteroposterior radiographs of the knee showing the injury (left) and post-reduction normal configuration (right). “Red dot” shows that the abnormality was detected by the attending radiographer.

**Figure 8.**
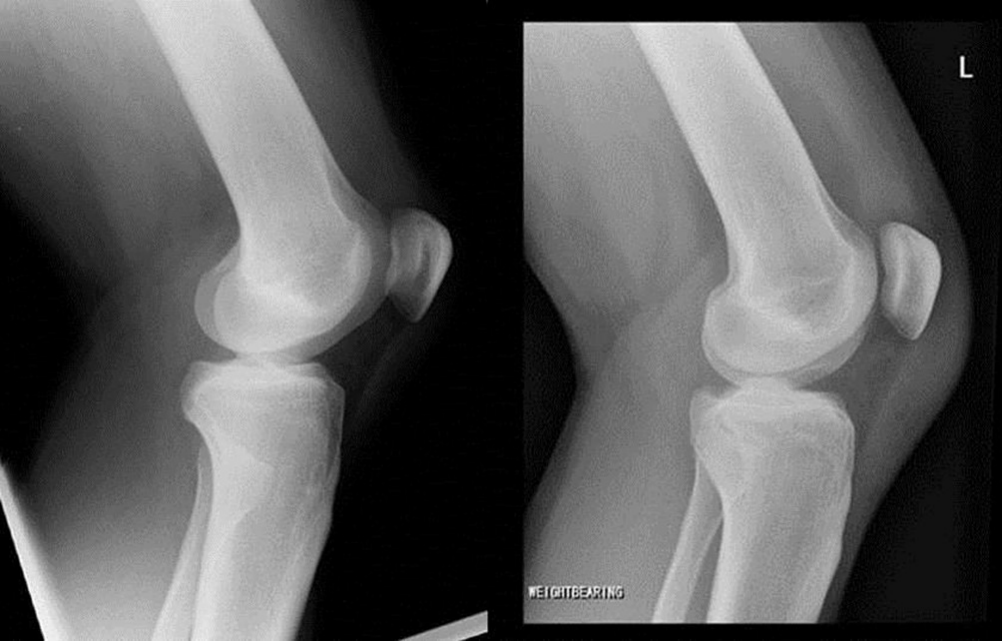
Lateral radiographs of the knee showing the injury (left) and post-reduction normal configuration (right).

Radiographic findings in PTFD are shown in Figures 7 and 8. In this case, PTFD is suggested by lateral translation of the fibular head in relation to the tibia on what remains an AP radiograph of the knee.

Adequate plain radiograph imaging for this condition is as follows:

- AP radiograph—adequate exposure, patella centralized over the femoral condyles, with the beam centred 2 cm below the apex of the patella and at 90° to the long axis of the tibia.^16^
- Lateral radiograph—adequate exposure, patella projected clear of the femur, femoral condyles congruous and the proximal tibiofibular joint should not be visible.^16^
- Contralateral knee radiographs to permit comparison.

Should that not be possible, axial imaging is usually diagnostic in the form of CT scan or MRI. This can be particularly useful in cases of subtle patellofemoral joint dislocation.^13,17^

Management is with closed reduction, which should be completed in flexion, with force applied in the direction opposite to that of the dislocation of the fibular head. Should this not be possible, open reduction is required, although this is uncommon.

Complications of the injury include nerve injury, recurrence and secondary arthritis.^10,12^ Arthritis is rare owing to the relative stability of the joint. Recurrence can occur and would be one of the indications for considering surgical management. Another clear indication is the inability to perform a closed reduction. Operative management could include Kirschner wire fixation, joint fusion or proximal fibular excision.^18^ Nerve injury is seen most commonly with posteromedial fibular dislocations and should encourage prompt reduction to minimize long-term peroneal nerve palsy. Should the neurological symptoms not settle following reduction, urgent peroneal nerve exploration should be performed.

## Learning points

1. Tibiofibular dislocation is a recognized injury that usually happens in conjunction with major limb injuries.
2. Isolated tibiofibular dislocation is much less common, but still reported.
3. Diagnosis is made with clinical examination and plain radiographs. If there is diagnostic doubt, axial imaging in the form of CT scan or MRI can be used.
4. MRI permits examination of the common peroneal nerve, which can be injured, particularly in posterior fibular dislocation.
5. Management is ideally closed reduction under sedation or, if required, general anaesthesia.

## Consent

Written informed consent was obtained from the patient for publication of this case report, including accompanying images.
